# Localized Delivery of Interferon-β by *Lactobacillus* Exacerbates Experimental Colitis

**DOI:** 10.1371/journal.pone.0016967

**Published:** 2011-02-18

**Authors:** Adelle P. McFarland, Ram Savan, Sagie Wagage, Augustina Addison, Karthika Ramakrishnan, Megan Karwan, Tri Duong, Howard A. Young

**Affiliations:** 1 Cancer and Inflammation Program, Center for Cancer Research, National Cancer Institute, Frederick, Maryland, United States of America; 2 Department of Food, Bioprocessing and Nutrition Sciences, North Carolina State University, Raleigh, North Carolina, United States of America; Centre de Recherche Public de la Santé (CRP-Santé), Luxembourg

## Abstract

**Background:**

There have been conflicting reports of the role of Type I interferons (IFN) in inflammatory bowel disease (IBD). Clinical trials have shown potent efficacy of systemic interferon-beta (IFN-β) in inducing remission of ulcerative colitis. Likewise, IFNAR1^−/−^ mice display an increased sensitivity to dextran sulfate sodium (DSS)-induced colitis, suggesting Type I IFN play a protective role during inflammation of the gut. Curiously, however, there have also been reports detailing the spontaneous development of IBD in patients receiving systemic IFN-β therapy for multiple sclerosis or hepatitis.

**Methodology/Principal Findings:**

To investigate the effects of local administration of IFN-β on a murine model of colitis, we developed a transgenic *Lactobacillus acidophilus* strain that constitutively expresses IFN-β (La-IFN-β). While pretreatment of mice with control *Lactobacillus* (La-EV) provided slight protective benefits, La-IFN-β increased sensitivity to DSS. Analysis showed colitic mice pretreated with La-IFN-β had increased production of TNF-α, IFN-γ, IL-17A and IL-13 by intestinal tissues and decreased regulatory T cells (Tregs) in their small intestine. Examination of CD103^+^ dendritic cells (DCs) in the Peyer's patches revealed that IFNAR1 expression was dramatically reduced by La-IFN-β. Similarly, bone marrow-derived DCs matured with La-IFN-β experienced a 3-fold reduction of IFNAR1 and were impaired in their ability to induce Tregs.

**Conclusions/Significance:**

Our IFNAR1 expression data identifies a correlation between the loss/downregulation of IFNAR1 on DCs and exacerbation of colitis. Our data show that *Lactobacillus* secreting IFN-β has an immunological effect that in our model results in the exacerbation of colitis. This study underscores that the selection of therapeutics delivered by a bacterial vehicle must take into consideration the simultaneous effects of the vehicle itself.

## Introduction

Type I interferons (IFN), including IFN-α and IFN-β, are widely expressed cytokines involved in innate anti-viral immune responses. Additionally, these cytokines have an immunomodulatory role in the anti-inflammatory host response. Type I IFN signal through a heterodimeric receptor complex comprised of two subunits, IFNAR1 and IFNAR2. When IFN-α/β bind to the IFNAR1/2 complex, there is recruitment and subsequent phosphorylation of STAT1 and STAT2 [1].

The anti-inflammatory properties of Type I IFN have been recognized in human diseases, such as multiple sclerosis (MS) [2]. Administration of Type I IFN in mouse models leads to reduced skin reactivity to lipopolysaccharides (LPS), reduced response to ischemic insult/chemokine injection, and protection from collagen-induced arthritis (CIA), experimental autoimmune encephalomyelitis (EAE) and delayed-type hypersensitivity-allotransplant rejection (reviewed in [3]). In the CIA model, IFN-β prevented the onset of arthritis or ameliorated existing disease by suppressing the proinflammatory mediators IFN-γ, IL-6, IL-12 and TNF-α and by inducing the anti-inflammatory cytokine IL-10 [4], [5]. Additionally, Type I IFN can suppress inflammatory responses by stimulating tristetraprolin, a strong suppressor of TNF-α and IFN-γ [6], [7], or by inhibiting CXCL8, a chemokine that recruits neutrophils and leukocytes to areas of inflammation [8], [9].

The anti-inflammatory properties of IFN-β are perhaps best recognized and studied in MS, a chronic inflammatory disorder of the central nervous system. As a standard therapy for relapsing remitting MS, the mechanisms of IFN-β action have been widely studied. It has been shown that IFN-β inhibits expression of adhesion molecules on the surface of endothelial cells and may lead to reduced adhesion of leukocytes and attenuation of inflammatory reactions [10], [11]. Additionally, IFN-β is known to downregulate matrix metalloproteinases that are implicated in the immunopathogenesis of MS [12], [13]. Although Th1 cells are involved in the autoimmune inflammation in MS and in the mouse model EAE, IL-17-secreting T cells have been shown to play a pivotal role in the pathogenesis of the disease [14]–[16]. Recently, studies have shown that IFN-β inhibits Th17 cell differentiation, which may help explain its therapeutic benefits in MS [17]–[19].

The anti-inflammatory abilities of Type I IFN have also been investigated in ulcerative colitis (UC), an inflammatory bowel disease (IBD) that causes ulcers in the lining of the colon and rectum. There have been several clinical trials in UC patients that have shown efficacy of systemic administration of IFN-β, with up to 88% remission in patients with steroid-refractory UC [20]–[22]. Curiously, there have been reports of exacerbation or spontaneous development of IBD, such as Crohn's disease and UC, in patients receiving IFN-α/β therapy for chronic hepatitis or MS [23]–[27]. Thus, the mechanism mediating this paradoxical effect of IFN-β *in vivo* warrants further investigation.

In recent years, research into the pathogenesis of gut inflammation has revealed Th17 cells as central effectors that produce proinflammatory cytokines including IL-17 [28]–[30]. Given the ability of IFN-β to inhibit Th17 cells in EAE, there is a need to investigate the role of IFN-β in Th17-mediated gut inflammation. One study showed that in dextran sulfate sodium (DSS)-induced colitis, IFN-β offers protective benefits when induced via TLR9 and when administered in recombinant form during the course of colitis [31], although the effect on IL-17 expression was not reported. One human UC study showed a correlation between response to Type I IFN therapy and suppression of IL-17 production and Th17 differentiation [32]. However, there is a lack of understanding of how IFN-β may abrogate intestinal inflammation and whether it can specifically counteract Th17 in this context.

Based on its anti-inflammatory properties and previous success in colitis studies, we investigated the effects of IFN-β in a murine colitis model. Systemic administration of IFN-β often results in flu-like symptoms and is generally not well tolerated. Previous work has shown efficacy in localized delivery of therapeutics in colitis models via lactic acid bacteria [33]–[35]. This approach avoids systemic toxicity associated with cytokine-based therapies and offers delivery directly to inflamed mucosal tissue. In this study, we developed a genetically modified bacterium, *Lactobacillus acidophilus*, that constitutively expresses IFN-β. Using these transgenic bacteria, we employed a pretreatment model of acute DSS-induced colitis where mice were administered *Lactobacillus* secreting IFN-β (La-IFN-β) or an empty vector control (La-EV). Surprisingly, our data show that pretreatment with La-IFN-β exacerbates DSS colitis, with an increase in TNF-α, IFN-γ, IL-17A and IL-13 production by intestinal tissues over the controls.

## Materials and Methods

### Cells and mice

Ethics statement: Mice were bred and maintained in specific pathogen-free conditions in accordance with the procedures outlined in the “Guide for Care and Use of Laboratory Animals” (National Institute of Health, Bethesda, MD, 1996). All animal studies were approved by the Animal Care and Use Committee under the protocol ID 09-027 at the National Cancer Institute, NIH USA. A/JCr, C57BL/6Ncr and BALB/cAnNcr mice were purchased from Charles River Laboratories (Wilmington, MA). IFN-α/β receptor 1 (IFNAR1)-deficient mice (BALB/c-alpha-R KO) were provided by Joan Durbin (Ohio State University, Columbus, OH). All mice were analyzed between 8–12 weeks of age. The C57BL/6 murine macrophage cell line was derived as previously described [36] and grown in DMEM (BioWhittaker, Walkersville, MD) supplemented with 10% heat-inactivated fetal bovine serum (FBS; Atlanta Biologicals, Lawrenceville, GA), penicillin (100 U/ml), streptomycin (100 µg/ml) and L-glutamine.

### Expression vector and bacteria

*Lactobacillus acidophilus* NCK 1887 containing the vector pTRK926 (termed La-IFN-β), which constitutively expresses murine IFN-β, was used as a localized delivery method of IFN-β to mice prior to induction of DSS colitis. *L. acidophilus* NCK 1895 containing the vector pTRK882 [37] (termed La-EV), which does not have the murine IFN-β gene inserted, was used as an empty vector control. The murine IFN-β gene was codon optimized (website: www.jcat.de/) for expression in *Lactobacillus* and then cloned into the pTRK882 plasmid to generate pTRK926. The expression of IFN-β in this vector is under the control of the constitutive high-expressing promoter P*_pgm_*. *Lactobacillus* cultures were grown statically in sealed conical tubes in deMann, Rogosa, and Sharpe (MRS) broth (BD Difco, Franklin Lakes, NJ) at 37°C, with 5 µg/ml of erythromycin (Sigma-Aldrich). Solid media for plating was prepared with 1.5% agar (BD Difco) and 5 µg/ml of erythromycin. Bacterial plates containing *Lactobacillus* were grown in anaerobic bags for 48 h (Bio-Bag Type A; BD Biosciences, San Jose, CA). For gastric gavage of mice, bacteria were grown until log-phase (based on growth curves), washed twice with PBS and resuspended to achieve 4×10^9^ CFU/ml. Mice were subjected to gastric gavage with 250 µl of PBS or the bacterial strains (1×10^9^ CFU/mouse/gavage). Quantification of bacterial CFU was performed on probiotic preparations by plate count method, and mouse feces were checked daily for the presence of the transgenic *Lactobacillus*.

### Bone-marrow derived dendritic cell experiments

Murine bone marrow-derived dendritic cells (BMDCs) were generated from mice as previously described [38]. Briefly, bone marrow cells were grown in RPMI 1640 (BioWhittaker) supplemented with 10% heat-inactivated FBS, penicillin (100 U/ml), streptomycin (100 µg/ml), L-glutamine, 1 mM sodium pyruvate, 1 mM NEAA, 10 mM HEPES, 50 µM β-mercaptaethanol, IL-4 (10 ng/ml) and GM-CSF (20 ng/ml). On day 2 following plating, non-adherent cells were removed from the culture. On day 4 cells were washed and plated in fresh media and on day 6 the BMDCs were harvested. BMDCs from wild-type or IFNAR1^−/−^ were matured with 1000 U/ml of IFN-β (PBL InterferonSource, Piscataway, NJ) or 100 ng/ml of LPS (Sigma-Aldrich, St. Louis, MO) for 24 h before analysis. BMDCs from A/J mice were matured with TNF-α (50 ng/ml), TNF-α + La-EV (100:1 ratio, La-EV:BMDCs) or TNF-α + La-IFN-β (100:1 ratio, La-IFN-β:BMDCs) for 48 h prior to co-culture with CD3/CD28 (1 µg/ml each; BioLegend, San Diego, CA) activated splenocytes at a 1∶2 ratio for 7 d.

### Western blot analysis

To check for IFN-β expression/secretion, supernatants of overnight bacterial cultures were collected and subjected to trichloroacetic acid (TCA) protein precipitation. Briefly, 0.5 µl of 100 mM PMSF, 1 µl of 1 M DTT and 300 µl of 100% TCA was added to 1.5 ml of cell-free bacterial supernatant, vortexed and held on ice for 30 min. Supernatants were then centrifuged at 4°C at 14,000 rpm for 5 min, washed twice with cold acetone and dried on a 95°C heat block for 5 min. 1 µl of 1 M DTT, 5 µl of LDS sample buffer (Invitrogen, Carlsbad, CA) and 19 µl of PBS were added to the pellet and boiled for 10 min. Samples were run on an SDS–polyacrylamide (Invitrogen) gel and transferred to Immobilon-P membranes (Millipore, Billerica, MA). The membranes were blocked at room temperature in 5% milk/Tris-buffered saline Tween 20 solution. The membranes were then probed with anti-murine IFN-β (1∶1000; BioLegend) and developed with HRP substrate (Amersham Biosciences, Piscataway, NJ).

### DSS-induced colitis model

C57BL/6Ncr and A/J mice were given 3% DSS (MW 36,000–50,000; MP Biomedicals, Solon, OH) dissolved in reverse osmosis water ad libitum for 7 d (i.e., one cycle of DSS). Gastric gavages of PBS or bacterial treatments (1×10^9^ CFU/mouse/gavage) were given for three consecutive days prior to the administration of DSS. For quality control, cultures used for gavages were checked for the presence of IFN-β. On day 8, mice were returned to normal drinking water and sacrificed on day 10. Mice were weighed daily, checked for diarrhea and rectal prolapse/macroscopic bleeding. The colon was assessed macroscopically and given a semi-quantitative score (0–3) based on the degree and extent of mucosal hemorrhaging.

### Isolation of intestinal lymphocytes

Small intestinal intraepithelial lymphocytes (smIELs) were isolated as previously described [39]. Briefly, small intestines were harvested from mice, flushed and cleaned with HBSS (BioWhittaker) containing 2.5% FBS (complete media, CM). The Peyer's patches (PPs) were isolated from small intestines and mechanically processed through a 100 µm cell strainer (BD Biosciences) for a single cell suspension. Mesenteric lymph nodes (MLNs) were processed similarly. The small intestines (with Peyer's patches removed) were incubated in CM in the presence of 1 mM DTT (Sigma-Aldrich) at 37°C and 250 rpm for 20 min and subsequently in 1.3 mM EDTA (Boston BioProducts, Ashland, MA) in CM at 37°C for 1 h at 250 rpm. After each incubation the supernatant fraction containing the smIELs was strained through a 100 µm strainer and pooled. The cells were strained twice more through 40 µm strainers and washed in CM twice before staining for FACS analysis.

### Quantification of cytokine production in sera and colonic culture supernatants

Two cm of the distal colon was harvested from colitic mice, weighed and cultured overnight in RPMI 1640 supplemented with 10% heat-inactivated FBS, penicillin (100 U/ml), streptomycin (100 µg/ml), L-glutamine and Amphotericin B (1 µg/ml). Cytokine levels were standardized for 100 mg of colonic tissue. Murine IFN-γ, IL-6, IL-10, IL-12p70, IL-17A, MCP-1 and TNF-α were measured using a cytometric bead array (CBA) kit according to the manufacturer's instructions (BD Biosciences). BMDC cell-free supernatants were tested for murine IL-4 and IL-6 using a Th1/Th2 CBA kit according to the manufacturer's instructions (BD Biosciences). Murine IL-27p28 and IL-23 levels were determined in cell-free supernatants by a specific ELISA kit (Quantikine; R&D Systems).

### RNA isolation, RT-PCR and focused array

Sections of the distal colon were isolated from colitic mice and snap frozen in liquid nitrogen for RNA analysis. The tissue was dissociated with 1.0 mm glass beads using a mini-beadbeater (Biospec) in Trizol (Invitrogen) and RNA extraction was performed according to the manufacturer's instructions. The RNA was reverse transcribed to cDNA (Invitrogen) and relative quantification was performed by real-time PCR (RT-PCR) for IL-17A and Foxp3 using HPRT as endogenous control according to the manufacturer's instructions and analyzed by DataAssist V2 (ABI). A focused array (Lonza, Cologne, Germany; Mouse Cytokines and Receptors 00189699) was performed on colonic RNA according to the manufacturer's instructions and the results were analyzed using RealTime StatMiner software V4.0 (Integromics).

### Cell stimulations and Abs used for flow cytometry

Flow cytometry was performed on single cell suspensions using an LSRII (BD Biosciences). The following Abs for extracellular staining were purchased from eBioscience (San Diego, CA): CD45 (30-F11), CD3 (17A2), CD4 (GK1.5), CD25 (PC61.5), CD11c (N418) and CD103 (2E7). Additional Abs were purchased from BioLegend (San Diego, CA): I-A/I-E (M5/114.15.2), CD40 (3/23) and IFNAR1 (MAR1-5A3). CD11b (Mac1, M1/70) was purchased from BD Biosciences. For intracellular analysis of Foxp3, cells were fixed/permeabilized using the Foxp3 Staining Buffer Set (eBioscience) according to the manufacturer's instructions. Cells were stained for Foxp3 (FJK-16s; eBioscience). For intracellular IFN-γ cytokine analysis, cells were activated with 10 ng/ml PMA (Sigma-Alrich) and 1 µM/ml ionomycin (Sigma-Alrich) for 5 h in the presence of Brefeldin A (eBioscience) in RPMI 1640 supplemented with 10% heat-inactivated FBS, penicillin (100 U/ml), streptomycin (100 µg/ml) and L-glutamine. After stimulation, cells were fixed/permeabilized using a cytofix/cytoperm kit (BD Biosciences). Cells were stained for IFN-γ (DB-1; BioLegend). For intracellular analysis of STAT1 phosphorylation, C57BL/6 macrophage cells were fixed/permeabilized using paraformaldehyde/methanol and stained for Stat1 (pY701) (4a; BD Biosciences).

### Statistical analysis

All statistical analysis was performed using the GraphPad Prism software (version 5.00; GraphPad, San Diego, CA). Data are expressed as ± SEM. The Student two-tailed unpaired, parametric *t* test was used to assess statistical differences between two experimental groups. Asterisks indicate statistical differences, * *p*<0.05, ** *p*<0.01, *** *p*<0.001.

## Results

### IFNAR1^−/−^ mice are more susceptible to DSS-induced colitis, have less responsive DCs and decreased regulatory T cells in their Peyer's patches

**Figure 1:**
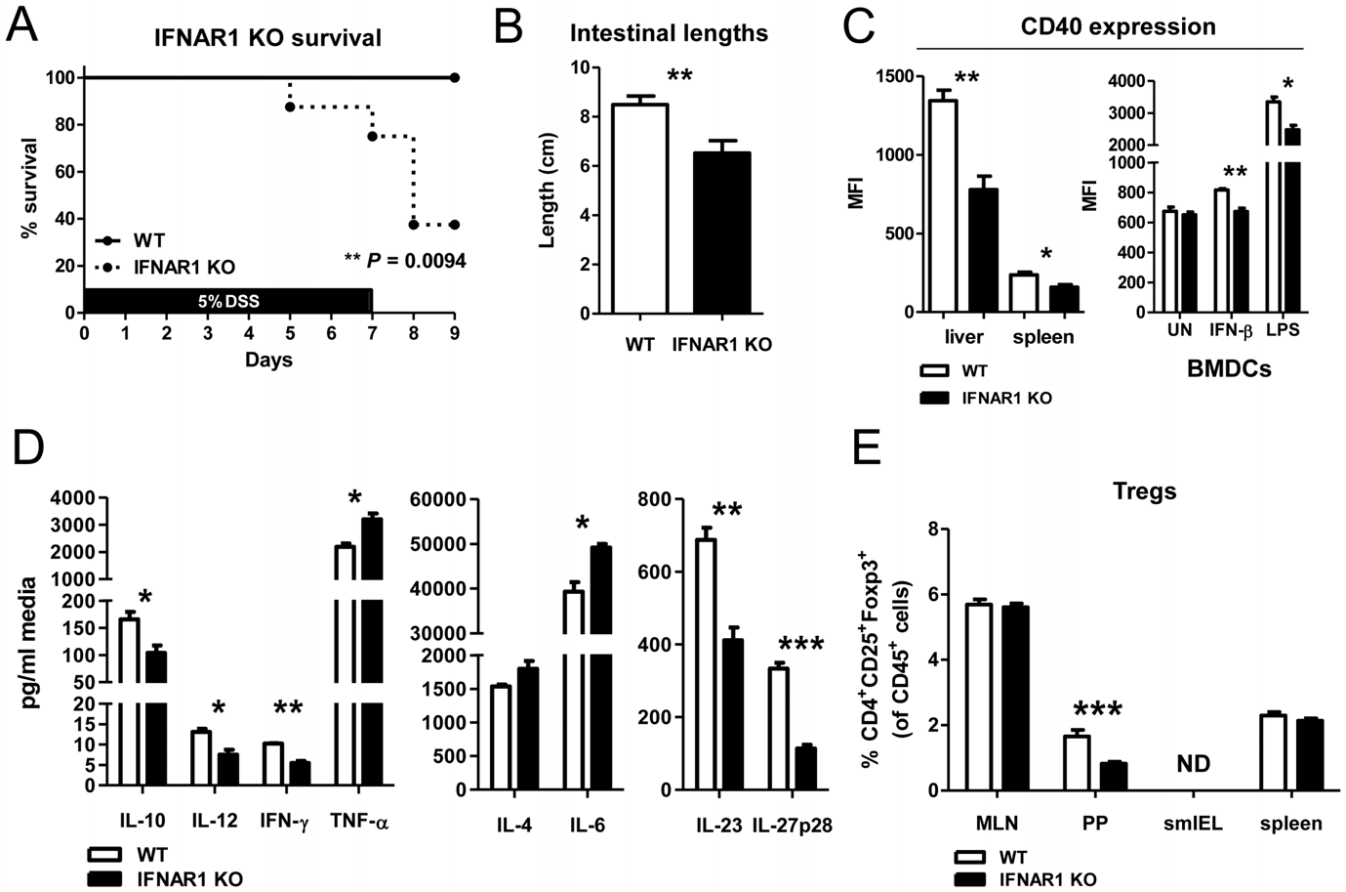
IFNAR1^−/−^ mice are more susceptible to DSS-induced colitis and have altered DC and Treg profiles. (**A, B**) IFNAR1^−/−^ mice experienced increased weight loss and greater degree of intestinal shortening when administered 5% DSS in their drinking water for 7 d compared to wild-type controls (n = 10). (**C**) DCs isolated from liver and spleen of IFNAR1^−/−^ mice and BMDCs matured with IFN-β (1000 U/ml) or LPS (100 ng/ml) for 24 h were analyzed for cell surface expression of CD40. DCs were gated as CD45^+^ClassII^+^CD11c^+^. (**D**) Levels of cytokines, measured by CBA or ELISA (IL-23 and IL-27p28), in BMDC supernatants stimulated with LPS (100 ng/ml) for 24 h from IFNAR1^−/−^ or wild-type mice (n = 5). (**E**) Percent Tregs, gated as CD45^+^CD4^+^CD25^+^Foxp3^+^, in MLNs, PPs, smIELs and spleens of IFNAR1^−/−^ and wild-type mice (ND = not detected, n = 10). The data is represented as the mean ± SEM. * *p*<0.05, ** *p*<0.01, *** *p*<0.001.

It has been reported that IFNAR1^−/−^ mice have increased susceptibility to DSS-induced colitis [31], [40]. To confirm these findings, we administered 5% DSS in the drinking water of BALB/c wild-type or IFNAR1^−/−^ mice for 7 d. Mortality and colonic shortening, which occurs as a result of inflammation in the mucosa and correlates with disease severity, were greatly increased in mice lacking IFNAR1 (Figure 1A and 1B).

In order to better understand how the lack of IFNAR1 contributes to increased intestinal inflammation, we examined the phenotype of dendritic cells (DCs) in IFNAR1^−/−^ mice as Type I IFN have been shown to play a critical role in the development and activation of DCs in steady state as well as inflammatory settings [41]–[43]. However, one caveat to this analysis is that these mice are systemically deficient in IFNAR1. To investigate the phenotype of DCs in mice lacking IFNAR1, cells from spleen and liver were isolated from wild-type and IFNAR1^−/−^ mice. Flow cytometric analysis showed that DCs, defined as ClassII^+^CD11c^+^, from IFNAR1^−/−^ had lower levels of surface expression of CD40 co-stimulatory molecules than wild-type controls (Figure 1C). BMDCs from IFNAR1^−/−^ mice matured with IFN-β or LPS also showed decreased surface expression of CD40 (Figure 1C). Following maturation with LPS, the BMDC culture supernatants were collected and assayed for cytokines. IFNAR1^−/−^ BMDCs produced lower levels of IL-10, IL-12, IL-23 and IL-27p28 than wild-type BMDCs, however they produced more TNF-α and IL-6 (Figure 1D). The decreased expression of co-stimulatory molecules, in addition to an overall decreased capacity to produce cytokines upon LPS stimulation, suggests that DCs from IFNAR1^−/−^ mice are functionally defective.

As DCs are important in the induction of regulatory T cells (Tregs) we investigated whether Treg populations are affected in IFNAR1^−/−^ mice. Lymphocytes from mesenteric lymph nodes (MLNs), Peyer's patches (PPs), small intestine (small intestinal intraepithelial lymphocytes [smIELs]) and spleen were isolated and analyzed by flow cytometry. Tregs were defined as CD4^+^CD25^+^Foxp3^+^. Strikingly, the IFNAR1^−/−^ mice had significantly decreased percentages of Tregs within the PPs specifically (Figure 1E). The functional changes in DCs combined with an altered intestinal Treg profile in the PPs of IFNAR1^−/−^ mice and an increased sensitivity to colitis suggest a role for Type I IFN signaling in controlling intestinal inflammation and homeostasis.

### Transgenic *L. acidophilus* expresses functional murine IFN-β

**Figure 2:**
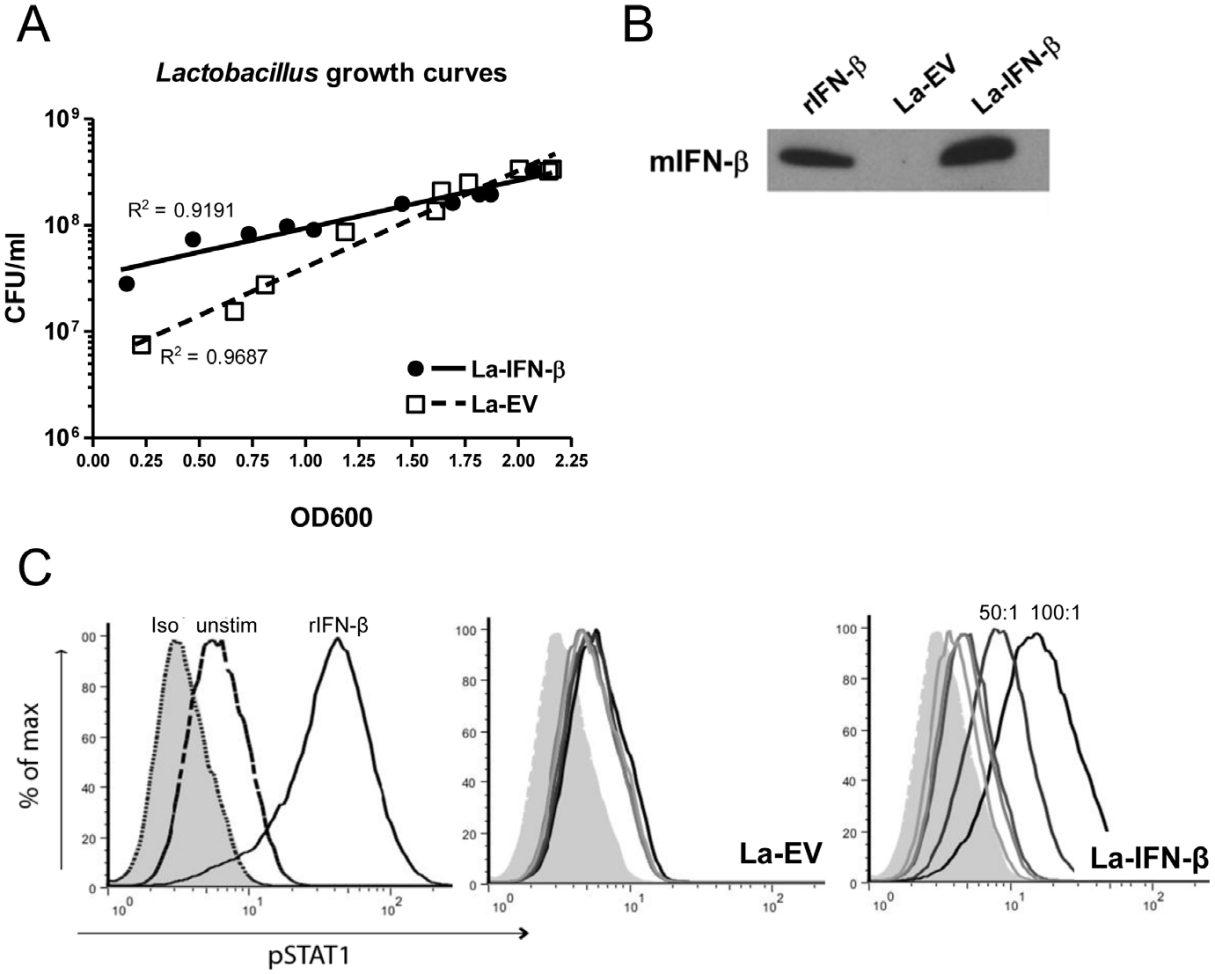
La-IFN-β expresses biologically functional murine IFN-β. (**A**) Growth curves for La-IFN-β and La-EV strains correlating CFU/ml of bacterial culture with OD600 values. (**B**) Western blot showing detection of murine IFN-β in the supernatant from an overnight culture of transgenic La-IFN-β. Prior to Western blotting, the protein was precipitated using TCA. Positive control is 100 U of recombinant murine IFN-β. (**C**) Phosphorylation of STAT1 in a C57BL/6 macrophage cell line after 60 min stimulation with rIFN-β (1000 U) as a positive control or co-culture with 5∶1, 10∶1, 50∶1 or 100∶1 ratios of La-EV or La-IFN-β.

The data from the IFNAR1^−/−^ mice indicates Type I IFN signaling is important for controlling intestinal inflammation. IFN-β is routinely administered as an anti-inflammatory therapy for MS, although systemic side effects are not well tolerated. The novel use of lactic acid bacteria to deliver therapeutics locally was first reported by Steidler and co-workers [34]. Using this technique to investigate the effect of local IFN-β administration on gut inflammation, we developed a transgenic *Lactobacillus* that constitutively expresses murine IFN-β (La-IFN-β) or a control strain (La-EV). We generated individual growth curves for each strain for quantification of bacterial colony forming units (CFU) and used these to determine resuspension volumes for *in vitro* and *in vivo* experiments (Figure 2A).

Expression and secretion of the IFN-β protein into bacterial supernatant was assessed by Western blot. IFN-β was detected in the media from the La-IFN-β culture but was absent in the La-EV control (Figure 2B). To evaluate the biological activity of the secreted IFN-β, we stimulated a C57BL/6 macrophage cell line with recombinant IFN-β as a control and observed rapid phosphorylation of STAT1 in a dose dependent manner (Figure S1). Co-culture of macrophage cells with La-IFN-β also resulted in rapid phosphorylation of STAT1 in a dose dependent manner, while no phosphorylation was observed in co-culture with the La-EV control in the time frame analyzed (Figure 2C). The activation of STAT1 by La-IFN-β indicates that the secreted IFN-β protein can bind IFNAR and initiate the STAT1 signaling pathway.

### Pretreatment with La-IFN-β exacerbates DSS-induced colitis

To investigate the efficacy of localized delivery of IFN-β, we used an acute model of colitis whereby 3% DSS was administered in the drinking water of mice for 7 d. Previous studies have demonstrated that systemic delivery of recombinant IFN-β during the course of DSS administration was sufficient to reduce colitis in mice [31], [40]. To study the protective capacity of La-IFN-β on DSS-induced gut inflammation, we employed a preventative model of therapy.

**Figure 3:**
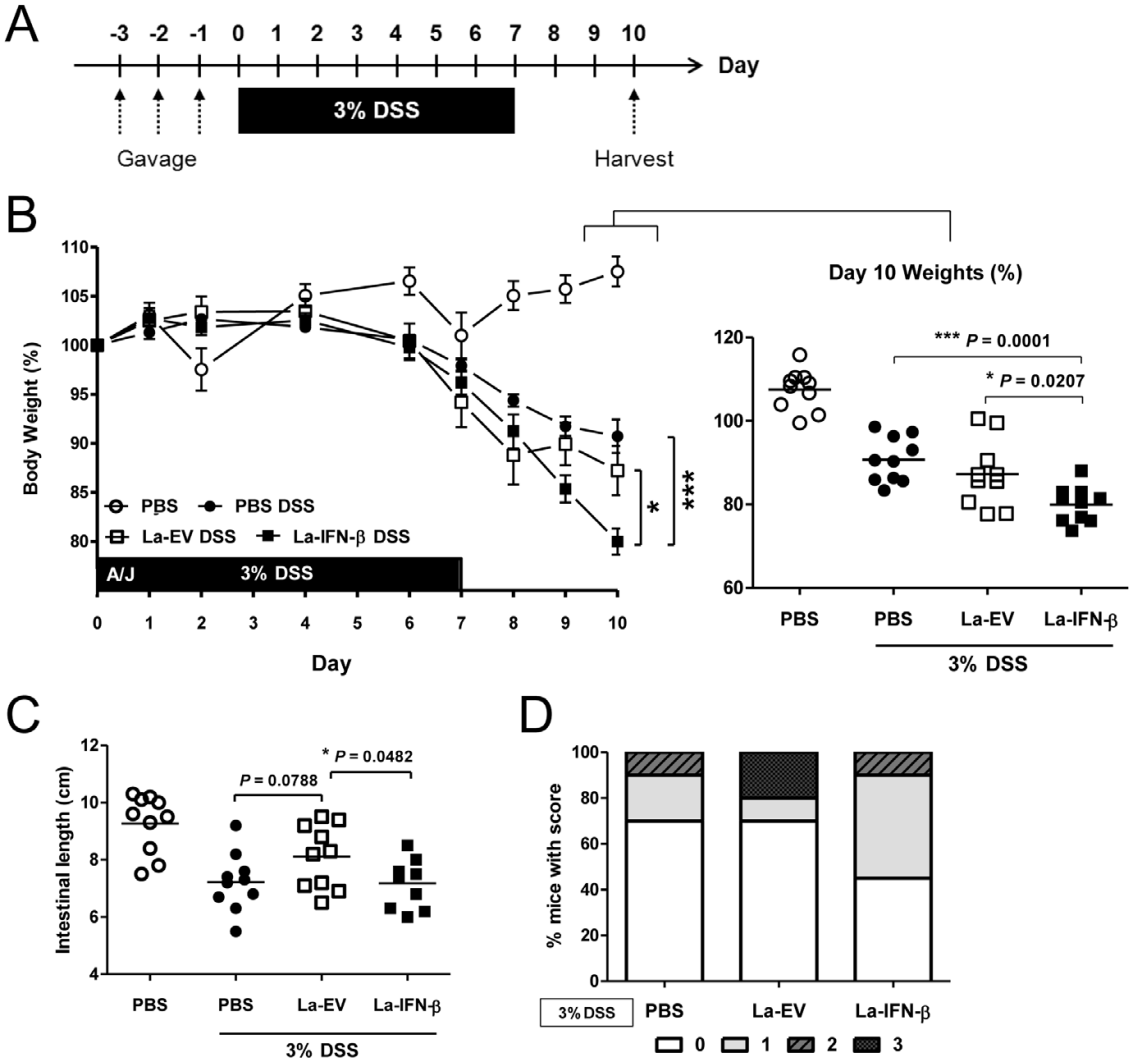
Pretreatment of mice with La-IFN-β exacerbates DSS-induced colitis. (**A**) On days -3, -2 and -1 mice were gastric gavaged with 10^9^ CFU of the bacterial treatments and days 0-7 3% DSS was administered in the drinking water. (**B**) Percent body weight loss of mice pretreated with La-IFN-β, La-EV or PBS for three days. The 100% body weights were taken as the weights of mice just prior to DSS administration. Day 10 percent body weights are plotted to show individual mice (n = 10). (**C**) Intestinal lengths (cm) of mice on day 10 following DSS administration. Colons were harvested and measured from the proximal end just below the cecum and to the anus (n = 10). (**D**) Following the harvest of the colons, the tissue was macroscopically examined for severity and distribution of intestinal hemorrhaging and scored on a scale from 0–3 (0 = no visible hemorrhaging, 1 = diffuse hemorrhaging, 2 = widespread hemorrhaging, 3 = severe hemorrhaging throughout colon). Results shown are representative of three independent experiments.

To determine the short-term duration of *Lactobacillus* colonization in mice, we delivered 10^9^ CFU of La-IFN-β or La-EV control by oral gavage for 3 consecutive days. For quality control, in all experiments involving gastric gavages the cultures were checked for the presence of IFN-β (Figure S2A). This dosing regimen resulted in transient colonization, with >10^7^ CFU of bacteria detected in the feces 4 d following the last gavage (data not shown). To investigate whether the transgenic La-IFN-β could prevent colitis induced by DSS, A/J mice were pretreated with La-IFN-β, La-EV or PBS as a control (Figure 3A). Pretreatment with La-EV showed no significant protective effect by gross morphological analysis over PBS, although there was a lesser degree of intestinal shortening (Figure 3C). Pretreatment with La-IFN-β, however, resulted in a marked worsening of colitis symptoms. Mice pretreated with La-IFN-β had significantly increased weight loss over both La-EV and PBS treated groups, greater intestinal shortening and an increased incidence of intestinal hemorrhaging (Figure 3B – 3D). The effect on weight loss by pretreatment with La-IFN-β was also observed in C57BL/6 mice, although the severity of colitis was worse in the more sensitive A/J strain (Figure S2B). Additionally, mice in the La-IFN-β group had the most pronounced incidence of diarrhea with visible hemoccult (data not shown). Taken together, these data demonstrate that pretreatment with La-IFN-β results in an increased sensitivity to and thereby exacerbation of DSS-induced colitis.

### Increased levels of proinflammatory cytokines in colitic mice pretreated with La-IFN-β

**Figure 4:**
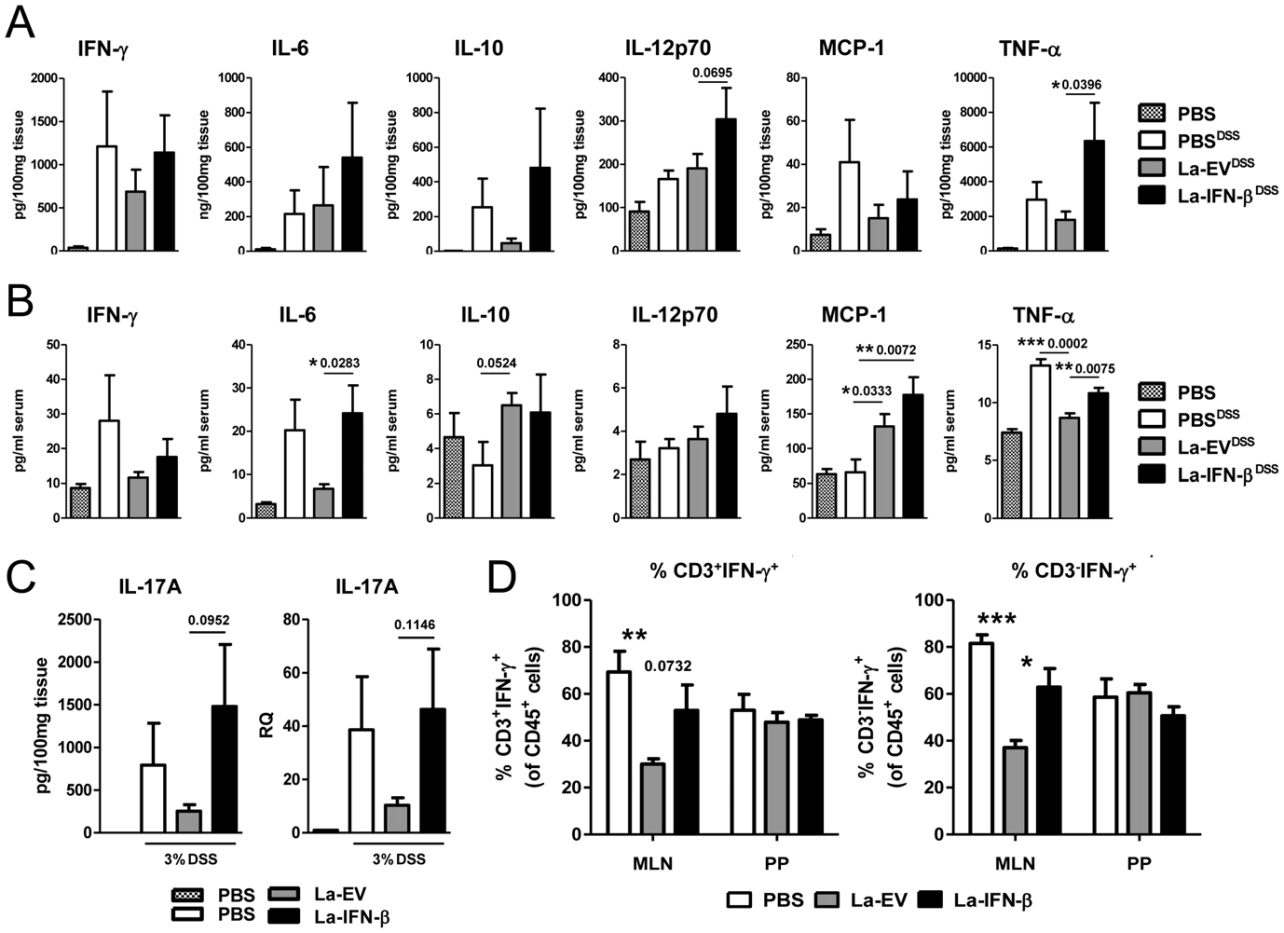
Elevated levels of inflammatory cytokines in mice pretreated with La-IFN-β over La-EV. (**A**) 2 cm of the distal colon was removed and weighed from colitic mice pretreated with PBS, La-EV or La-IFN-β (and a no DSS control group) and cultured overnight (n = 10). IFN-γ, IL-6, IL-10, IL-12p70, MCP-1 and TNF-α cytokine levels were assayed by CBA and standardized to concentration per 100 mg tissue. (**B**) Serum was assayed for the presence of inflammatory cytokines by CBA on day 7 of DSS administration (n = 5). (**C**) Measurement of IL-17A in colonic tissue culture (as outlined in Fig.4A) by CBA and the relative quantification of IL-17A mRNA in the distal colon was measured by RT-PCR. For RT-PCR, the colitic groups were compared relative to a no DSS control (RQ = 1) and HRPT was used as an endogenous control. (**D**) Cells isolated from the MLNs and PPs of colitic mice were stimulated with PMA (10 ng/ml) and ionomycin (1 µM/ml) for 5 h with the addition of Brefeldin A after 2 h. The cells were then stained for CD3 and fixed for intracellular detection of IFN-γ. Data is presented as %CD3^+^IFN-γ^+^ or %CD3^−^IFN-γ^+^ cells in the CD45^+^ population. The data is represented as the mean ± SEM. * *p*<0.05, ** *p*<0.01, *** *p*<0.001.

To study the inflammatory profile of colitic mice pretreated with PBS, La-EV or La-IFN-β, intestinal cytokine levels were assessed. Overnight cultures of distal colonic explants were assayed for cytokine protein levels by CBA. Among those tested, IL-12p70, TNF-α and IL-17A were increased in mice pretreated with La-IFN-β compared to the La-EV group (Figure 4A and 4C). Additionally, serum cytokine levels demonstrated significant differences in TNF-α, MCP-1 and IL-6 (Figure 4B). To determine whether differences in IL-17A could be detected at the mRNA level, RNA was extracted from the last 2 cm of the distal colon, the site where colitis inflammation is the most severe. The RT-PCR data show that there is an elevation of IL-17A mRNA in the colons of La-IFN-β pretreated mice over the La-EV controls, consistent with the data obtained from CBA (Figure 4C).

To investigate the ability of cells from the colitic mice to produce IFN-γ, we isolated lymphocytes from MLNs and PPs and stimulated with PMA and ionomycin for 5 h, with the addition of Brefeldin A after 2 h. The cells were then stained for CD3 and IFN-γ and analyzed by flow cytometry. Both CD3^+^ and CD3^−^ populations from the MLNs and PPs had significant levels of intracellular IFN-γ. Pretreatment with La-EV significantly reduced the levels of CD3^+^IFN-γ^+^ and CD3^−^IFN-γ^+^ cells in the MLNs compared to PBS, whereas La-IFN-β resulted in an increase over La-EV (Figure 4D). Overall, intracellular staining of lymphocytes revealed that the La-EV pretreatment reduced IFN-γ^+^ cells in the MLNs while La-IFN-β increased IFN-γ^+^ cells compared to La-EV.

The RNA isolated from the distal colon was analyzed in a focused array for cytokines and cytokine receptors. Using RealTime StatMiner software, the data was analyzed as genes significantly (*p<0.1) dysregulated in the La-EV and La-IFN-β pretreatments compared to the PBS control and plotted according to log fold change (Table 1). 3 genes were exclusively affected by La-EV compared to 34 by La-IFN-β and there were 12 genes which were common to both groups. Of the genes which were upregulated in both the La-EV and La-IFN-β groups, IL-28B and IFN-β had the highest log fold increases, although La-IFN-β had a much stronger effect. Additionally, IFN-α was the highest gene exclusively upregulated by La-EV. This data confirms previous studies which have shown that probiotics induce Type I IFN via TLR9 signaling [31], [44]. It also demonstrates the induction of high levels of Type I IFN in our system by both treatments. There were many inflammatory cytokines upregulated exclusively by La-IFN-β. Those that were upregulated by 1 log fold or higher include IL-9, IL-13, IL-4, IL-17A, IL-5, IL-24, IFN-γ, IL-10, IL-11, and IL-27, many of which are known cytokines involved in the pathogenesis of colitis. This upregulation is in comparison to PBS treated colitic mice and thus, demonstrates profound induction of these genes by La-IFN-β in this colitis model. The >2.5 log fold increase of IFN-α and IFN-β by La-EV verifies a role for Type I IFN in the protection conferred by this probiotic treatment and supports our initial hypothesis that IFN-β may aid in protection from colitis.

**Table 1:** Genes significantly* dysregulated compared to PBS control.

La-EV only genes			Shared genes					
*Gene*	Log fold	*p*-value	La-EV			La-IFN-β		
*Ifna1*	2.826	0.059	***Gene***	**Log fold**	***p*** **-value**	***Gene***	**Log fold**	***p*** **-value**
*Il4ra*	−2.323	0.059	*Il28b*	2.899	0.059	*Il28b*	3.203	0.006
*Ccr7*	−1.690	0.059	*Ifnb1*	2.572	0.096	*Ifnb1*	3.041	0.012
			*Il3*	2.286	0.069	*Il3*	2.437	0.012
**La-IFN-β only genes**			*Il19*	1.854	0.059	*Ccr3*	2.254	0.006
***Gene***	**Log fold**	***p*** **-value**	*Ccr3*	1.834	0.059	*Il2*	2.176	0.006
*Il9*	3.120	0.011	*Il23r*	1.725	0.059	*Il23r*	2.102	0.006
*Il13*	2.682	0.020	*Il25*	1.474	0.069	*Il12b*	1.961	0.006
*Il18r1*	2.109	0.014	*Il2*	1.420	0.067	*Il25*	1.891	0.006
*Il9r*	1.906	0.010	*Il12b*	1.357	0.069	*Il17f*	1.666	0.010
*Il4*	1.842	0.010	*Il6*	1.354	0.096	*Il6*	1.511	0.014
*Il17a*	1.827	0.013	*Il17f*	1.218	0.069	*Il19*	1.484	0.034
*Il5ra*	1.776	0.014	*Il11ra1*	−1.317	0.069	*Il11ra1*	−0.784	0.078
*Il5*	1.692	0.010						
*Cxcr2*	1.606	0.010						
*Ccr8*	1.540	0.010						
*Il24*	1.432	0.010						
*Cxcl2*	1.423	0.012						
*Ifng*	1.413	0.013						
*Il21*	1.364	0.020						
*Csf3*	1.300	0.014						
*Il10*	1.266	0.014						
*Il6ra*	1.228	0.014						
*Il11*	1.217	0.014						
*Il27*	1.186	0.024						
*Ccl1*	1.112	0.019						
*Ccr1*	1.108	0.029						
*Ccr4*	1.051	0.025						
*Il2ra*	0.902	0.074						
*Lifr*	0.791	0.073						
*Ccr5*	0.783	0.066						
*Csf2*	0.699	0.097						
*Il1a*	0.679	0.090						
*Tgfb1*	−0.735	0.082						
*Il1rn*	−0.751	0.079						
*Ccl28*	−0.764	0.091						
*Il10rb*	−0.783	0.086						
*Il18*	−0.792	0.086						
*Il6st*	−1.258	0.014						

Shown are the fold changes in genes which were significantly* (p<0.10) dysregulated in colitic mice (n = 3/group) pretreated with La-EV or La-IFN-β compared to the PBS control.

### IFNAR1 is differentially expressed on CD103 DCs in the gut

**Figure 5:**
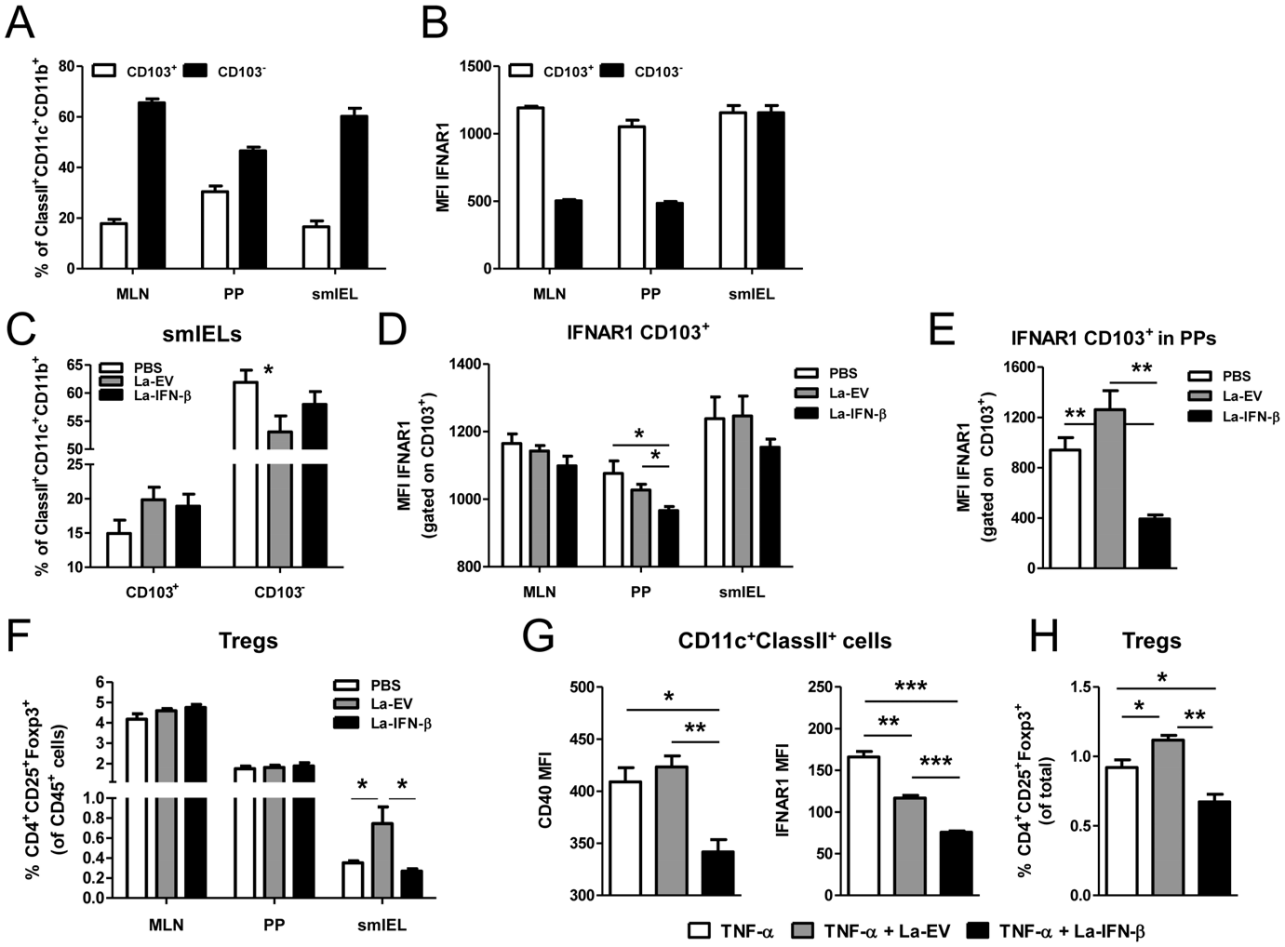
IFNAR1 is decreased on CD103^+^ DCs in the PPs of mice following treatment with La-IFN-β. (**A**) Percent distribution of CD103^+^ versus CD103^−^ DCs in the MLNs, PPs and smIELs of wild-type mice (n = 5) gated on CD45^+^ClassII^+^CD11c^+^CD11b^+^ cells. (**B**) IFNAR1 expression (mean fluorescence intensity, MFI) on CD103^+^ and CD103^−^ DCs in the MLNs, PPs and smIELs from wild-type mice. (**C**) CD103^+^ and CD103^−^ DC percentages in the smIELs of colitic mice pretreated with PBS, La-EV or La-IFN-β (n = 5). (**D**) IFNAR1 expression (MFI) on CD103^+^ DCs in the MLNs, PPs and smIELs of colitic mice in the different treatment groups. (**E**) IFNAR1 expression on CD103^+^ DCs in the PPs of healthy mice following three consecutive days of gastric gavage with PBS, La-EV or La-IFN-β. (**F**) Cells were isolated from colitic mice pretreated with PBS, La-EV or La-IFN-β (n = 5) from the MLNs, PPs and smIELs and stained for the presence of Tregs, which were gated as CD45^+^CD4^+^CD25^+^Foxp3^+^. The graph shows the % Tregs present in the CD45^+^ population. (**G, H**) BMDCs from wild-type mice were matured with TNF-α (50 ng/ml), TNF-α + La-EV or TNF-α+ La-IFN-β (100∶1 ratio, bacteria:BMDCs) for 2 d. The mature BMDCs were then co-cultured with CD3/CD28 (1 µg/ml) activated splenocytes for 7 d (1∶2 ratio, BMDCs:splenocytes). Following co-culture, DCs (gated as ClassII^+^CD11c^+^) were stained for CD40 and IFNAR1 expression (**G**) and the percent Tregs (CD4^+^CD25^+^Foxp3^+^) was determined (**H**). The data is represented as the mean ± SEM. * *p*<0.05, ** *p*<0.01, *** *p*<0.001.

Previous reports have demonstrated the capacity of CD103^+^ DCs in the gut to drive expansion of Tregs whereas CD103^−^ DCs are responsible for driving Th17 development [45]–[48]. As IFNAR1 is important in DC maturation and function, we sought to first investigate the distribution of CD103^+^ and CD103^−^ DC populations in steady state conditions. We performed isolations of lymphocytes from MLNs, PPs and smIELs and analyzed the DC populations by flow cytometry. DCs were gated as CD45^+^ClassII^+^CD11c^+^CD11b^+^ and then percentages of CD103^+^ and CD103^−^ cells were determined (Figure S3A). The population distribution of CD103^+^ and CD103^−^ DCs in the MLNs and smIELs were similar, with a 3 fold higher percentage of CD103^−^ DCs than CD103^+^ (Figure 5A). The PPs had the highest percentage of CD103^+^ DCs compared with the other tissues, but the CD103^−^ population was still higher than CD103^+^ as in the MLNs and smIELs. When we determined the mean fluorescence intensity (MFI) of IFNAR1 expression on the CD103^+^ and CD103^−^ populations, we discovered that the CD103^+^ DCs had a 2 fold higher expression of IFNAR1 compared with CD103^−^ DCs in MLNs and PPs (Figure 5B). However, IFNAR1 expression on CD103^+^ and CD103^−^ DCs was equivalent in the smIELs.

### IFNAR1 expression is decreased on CD103^+^ DCs in colitic mice pretreated with La-IFN-β

To determine whether La-EV or La-IFN-β treatment has an effect on CD103 DC populations, we analyzed DCs isolated from colitic mice. The percentage of CD103^+^ DCs was slightly increased in the La-EV group in the smIELs (Figure 5C) but was unchanged in the MLNs and PPs (data not shown). Additionally, there was a significant decrease in CD103^−^ DCs in the smIELs with La-EV and a corresponding slight increase with La-IFN-β pretreatment. When we looked at IFNAR1 expression on these DC subsets, we found that IFNAR1 was significantly decreased on CD103^+^ DCs in the PPs in the La-IFN-β group compared with both PBS and La-EV (Figure 5D). Furthermore, there was a trend towards decreasing IFNAR1 expression in the MLNs and smIELs on CD103^+^ DCs as well in mice pretreated with La-IFN-β. We observed no differences in IFNAR1 expression on CD103^−^ DCs (data not shown).

As the colitic mice were pretreated with the bacteria 10 d prior to the analysis of the DCs in the gut, we wanted to see the effect on IFNAR1 expression directly after the mice were gavaged. Groups of healthy mice were dosed with PBS, La-EV or La-IFN-β for 3 consecutive days and the CD103 DC populations in the MLNs, PPs and smIELs were analyzed on day 4. We observed no differences in the percentages of the CD103 populations in any of the tissues analyzed (data not shown). However, when we examined the levels of IFNAR1 expression in the groups, we saw a striking decrease in the receptor expression on CD103^+^ DCs in the PPs of mice treated with La-IFN-β (Figure 5E). This data provides a snapshot of what the receptor expression would have been on CD103^+^ DCs in the PPs just prior to initiating colitis. Furthermore, it shows that from just after bacterial treatment through the course of colitis, the IFNAR1 expression remained significantly lower on the CD103^+^ DCs in the PPs in the La-IFN-β mice than in the other groups.

Our previous data showed a link between the lack of IFNAR1 and the reduction of Tregs in the PPs (Figure 1E). Thus, we examined whether the pretreatments had an effect on the Treg populations in the intestinal tissues of the colitic mice. There was no difference in the percentages of Tregs in the MLNs or PPs between groups. However, there was a significant increase in Tregs in the smIELs of mice pretreated with La-EV over PBS and a significant decrease in Tregs in the La-IFN-β group compared to La-EV (Figure 5F). As isolations of lymphocytes from the large intestines did not yield enough cells to carry flow cytometric analysis, we examined potential differences in Tregs in this tissue by performing RT-PCR for Foxp3. We did not detect any differences in Foxp3 mRNA between the treatment groups in the large intestines, although DSS itself caused a 4-6 fold increase (Figure S3B).

### Maturation of BMDCs with La-IFN-β results in significantly lower expression of IFNAR1 and CD40 as well as an impaired ability to induce Tregs

To investigate the direct effect that La-IFN-β has on DCs, we isolated bone marrow from A/J mice and derived DCs using IL-4 and GM-CSF. We then matured the BMDCs with TNF-α or a combination of TNF-α + La-EV or TNF-α + La-IFN-β for 2 d. The matured BMDCs were then co-cultured with CD3/CD28-activated splenocytes for 7 d at a ratio of 1∶2 (BMDCs:splenocytes). After co-culture, the cells were stained for analysis of DCs and Tregs. The BMDCs matured with TNF-α + La-IFN-β resulted in an increased percentage of CD11c^+^ClassII^+^ cells compared to TNF-α or TNF-α + La-EV (Figure S4). The expression of the co-stimulatory molecule CD40 was significantly reduced on BMDCs matured with TNF-α + La-IFN-β (Figure 5G). Interestingly, there was a dramatic decrease in IFNAR1 expression on the BMDCs matured with TNF-α + La-IFN-β. However, there was also a reduction by TNF-α + La-EV compared with TNF-α alone, most likely as a result of autocrine TLR-induced IFN-β signaling. The co-culture with the activated splenocytes yielded a significantly increased percentage of Tregs by TNF-α + La-EV-matured BMDCs, while there was a very significant reduction in the percentage of Tregs by TNF-α + La-IFN-β BMDCs (Figure 5H). Thus, although IFNAR1 was reduced by TNF-α + La-EV there was still a positive effect on the induction of Tregs while a greater loss of IFNAR1 induced by TNF-α + La-IFN-β had a negative effect on Tregs. This suggests that the robustness of IFN-β signaling and subsequent IFNAR1 depression are important factors in DC activation and function.

## Discussion

There is significant scientific data to support a role for the intestinal microflora in maintaining intestinal homeostasis and/or contributing to intestinal inflammation [49]–[51]. Probiotic therapies are being widely investigated for their ability to attenuate IBD, such as UC. Additionally, transgenic lactic acid bacteria have been used to study the localized effects of therapies on gut inflammation in experimental colitis models and have been tested in human clinical trials with some success [52].

In the intestinal tract, bacteria, such as *Lactococcus* sp. and *Lactobacillus* sp., can trigger TLRs on DCs which in turn induces Type I IFN. Blockade of either TLR9 signaling or IFNAR1 inhibits the protective capability of TLR9 agonists, such as probiotics, in murine colitis [31], [40], [44]. A previous study showed that pretreatment of mice with CpG-ODN, a TLR9 ligand, protected from DSS-induced colitis by the induction of Type I IFN [53]. The protective benefits of TLR9-induced Type I IFN are thought to be mainly mediated by TLR9 activation in conventional DCs (cDCs), as depletion of cDCs abolished the anti-inflammatory effects of pretreatment with CpG-ODN [31], [40]. Additionally, *L. acidophilus* has been shown to induce IFN-β production by DCs *in vitro* by a TLR2 dependent mechanism [54]. Moreover, it is well documented that probiotics, such as *L. acidophilus,* induce Type I IFN via TLR stimulation and studies in experimental colitis suggest that this provides a protective benefit.

Studies on mice lacking IFNAR1 have demonstrated the importance of Type I IFN in the *in vivo* turnover, proliferation and trafficking of DCs [55]. Furthermore, IFNAR1 defective DCs have a diminished ability to stimulate naïve T cell proliferation [56]. We have shown that splenic and hepatic IFNAR1^−/−^ DCs have lower expression of CD40 and that BMDCs derived from IFNAR1^−/−^ mice produce lower levels of cytokines upon LPS stimulation. Additionally, as we and others have shown, IFNAR1^−/−^ mice are more susceptible to DSS-induced colitis. The reasons behind this increased sensitivity still need to be more thoroughly examined, although we have shown in this study that IFNAR1^−/−^ mice have a significantly reduced percentage of Tregs in the Peyer's patches.

Given the protective benefits of TLR9-induced Type I IFN in experimental colitis and reported success of recombinant IFN-β therapy in human colitis, we have developed a transgenic *Lactobacillus* strain that constitutively expresses murine IFN-β. We investigated whether localized administration of IFN-β by this bacterium could protect from experimental colitis. Surprisingly, the data from our study indicate that La-IFN-β exacerbates DSS-induced colitis compared to La-EV and PBS. Pretreatment of mice with La-EV prior to the administration of DSS had a slight protective effect as demonstrated by reduced levels of proinflammatory cytokines and an increased percentage of Tregs in the smIELs compared to the PBS control group. Interestingly, La-EV induced high levels of IL-28B production in colonic tissue, in addition to IFN-α and IFN-β; induction of IL-28B by *Lactobacillus* has not yet been reported. Pretreatment with La-IFN-β, however, resulted in significantly increased weight loss, greater intestinal shortening and an increased incidence of intestinal hemorrhaging. Furthermore, La-IFN-β pretreated mice had increased levels of proinflammatory cytokines, such as IL-17A and IL-13, and a lower percentage of Tregs in the smIELs. Taken together, our data demonstrate that mice pretreated with La-IFN-β experienced increased sensitivity to DSS.

To further investigate why La-IFN-β pretreatment exacerbated colitis, we focused our attention on CD103 DCs. As mentioned, IFNAR1 signaling plays an important role in DC development and function. A recent study has shown that Type I IFN signaling on splenic CD8^+^ DCs, in particular, enhances the turnover of these cells *in vivo* [55]. The analogous DC subset found in the intestine is CD103^+^ DCs and recent reports have highlighted the importance of these cells in colitis. Within the gut, DCs are central mediators of T cell skewing and activation, and thus they are important in controlling gut-associated inflammation by inducing either inflammatory Th17 cells or Tregs. CD103^+^ DCs are important in driving Treg proliferation whereas CD103^−^ DCs are involved in the production of proinflammatory cytokines and Th17 development [57]–[60]. To investigate the role of Type I IFN on these subsets and whether they may be involved in the exacerbation of colitis seen in our model, we first isolated gut-associated lymphocytes from wild-type mice. We characterized the distribution of CD103^+^ and CD103^−^ DCs in the MLNs, PPs and smIELs. The CD103^−^ DC population was predominant in all of these tissues; however, there was a higher percentage of CD103^+^ DCs in the PPs compared to the MLNs and smIELs. Analysis of these populations in our DSS pretreatment model revealed that there was a slight increase of CD103^+^ DCs and a significant decrease of CD103^−^ DCs in the smIELs of mice treated with La-EV compared to the PBS control. This increase/decrease of CD103^+^ and CD103^−^ populations, respectively, in the smIELs in the La-EV group may be playing a role in the slight protective effects seen in our model because, notably, the smIELs was the only tissue we analyzed which showed an increase in Tregs by this treatment. However, we did not detect striking differences in the CD103 DC populations in the La-IFN-β group, suggesting perturbation of these DC subsets is not playing a role in the exacerbation by La-IFN-β.

The susceptibility of IFNAR1^−/−^ mice to DSS-induced colitis suggests that the expression of this receptor is important in protection against gut inflammation. Although we did not see a difference in the percent distributions of CD103 DCs populations in the La-IFN-β group, we sought to determine whether pretreatment might have an effect on the expression of IFNAR1. Interestingly, analysis of CD103 DCs in wild-type mice revealed that IFNAR1 is more highly expressed on CD103^+^ DCs in the MLNs and PPs than on CD103^−^ DCs, suggesting that IFNAR1 signaling may play a more important role for CD103^+^ DC function than CD103^−^ DCs in these tissues. Examination of IFNAR1 expression on CD103^+^ DCs from the treatment groups in our colitis model revealed that there was a reduction in receptor expression in mice pretreated with La-IFN-β in all of the intestinal tissues we examined, with the most significant reduction occurring in the PPs. Additionally, analysis of the CD103^+^ DCs in the PPs of healthy mice gavaged with the treatments also showed a significant reduction in IFNAR1 expression by La-IFN-β. As noted, of the gut populations we analyzed, the PPs of IFNAR1^−/−^ mice showed the most significant reduction in the percentage of Tregs, although we did not see differences in the percentage of Tregs in the PPs of the colitic mice at the time point we examined.

To further understand the effect of La-IFN-β on DCs and the subsequent effect on Tregs, we derived BMDCs and matured them with TNF-α, TNF-α + La-EV or TNF-α + La-IFN-β. The BMDCs matured with TNF-α + La-IFN-β showed a dramatic loss of IFNAR1 and, similar to the IFNAR1^−/−^ BMDCs we examined, had a marked reduction in the surface expression of CD40. When these BMDCs were co-cultured with activated splenocytes, we found that maturation with TNF-α + La-IFN-β significantly impaired those BMDCs ability to induce Tregs. Taken together, the IFNAR1 expression data from the colitic and healthy mice, as well as the effect of IFNAR1 loss or downregulation in IFNAR1^−/−^ mice or *in vitro* cultures respectively, suggest that the loss of IFNAR1 expression may correlate with a decreased ability to generate Tregs, which in the course of DSS colitis may ultimately lead to increased sensitization. It is known that the binding of Type I IFN with IFNAR1/2 results in internalization via endocytosis and degradation of the receptor [61], [62]. Interestingly, IFN-β forms a stronger and more stable complex with IFNAR1/2 and there is a 30-fold increase in complex lifetime over IFN-α [63], [64]. The engagement of IFNAR1 by IFN-β results in a transient decrease in surface expression as the receptors are internalized and degraded [65]. One study demonstrated that autocrine production of IFN-β from LPS and poly(I:C)-matured DCs caused a marked decline in the level of IFNAR1 lasting over 24 h and consequently, the cells lose their sensitivity to IFN-β stimulation [43]. Thus, the turnover rate of the receptor plays a critical role in the activity of IFN-β-stimulated DCs.

The signaling of the secreted IFN-β by La-IFN-β in addition to the high levels of Type I IFN induced by *Lactobacillus*, as seen in our expression data, may have resulted in an indirect suppression of IFNAR1 on CD103^+^ DCs in the PPs by a period of saturation with IFN-β in the environment. The decrease of IFNAR1 surface expression prior to induction of colitis may have inadvertently led to the inability of IFN-β to signal during the inflammatory period. Thus, the increased sensitivity of IFNAR1^−/−^ mice to DSS may, in effect, be mimicked in the La-IFN-β pretreated mice as a result of decreased receptor expression, which we observed both before and after DSS treatment. In support of this, we show that there is increased IL-17A both in colonic supernatants and at the mRNA level in the La-IFN-β group. Given that IFN-β has been shown to block the development of IL-17-producing T cells and expression of IL-17, these results indicate that IFN-β may not be signaling in the system.

Recently Mannon and co-workers demonstrated that UC patients who responded to IFN-β therapy had a marked reduction in IL-13 levels, both in peripheral blood and by lamina propria T cells [66]. IL-13, which is produced by both Th2 and NK T cells, has been shown to play a fundamental role in the pathogenesis of colitis and can be inhibited by Type I IFN [67]–[70]. In our gene expression data, IL-13 is upregulated 2.68 log fold in colitic mice pretreated with La-IFN-β, which again supports our conclusion that La-IFN-β exacerbates colitis and that this may be a result of the inability of IFN-β to signal. Interestingly, Mannon and co-workers show that non-response of UC patients to IFN-β therapy was associated with high baseline levels of IL-17. Taken together, the role of Type I IFN in controlling or exacerbating gut inflammation may be dependent upon factors intrinsic to the patients, such as IFNAR1 expression. Likewise, this may help to explain the spontaneous development of IBD in some patients receiving IFN-β therapy for MS or hepatitis. However, the underlying mechanisms for why decreased expression of IFNAR1 on DCs may promote exacerbation of colitis, whether it be through an impaired ability to induce Tregs or an inability to control proinflammatory cytokine expression, such as IL-17 and IL-13, and whether the level of receptor downregulation we observed has significant immunological effects needs to be investigated further. Additionally, it would be of interest to study the effects of La-IFN-β administration in other disease contexts, such as in a DSS treatment model or other models of colitis, or to see if the previously reported protective benefits of recombinant IFN-β in experimental colitis is dose dependent.

Here we have reported our finding that *Lactobacillus* expressing IFN-β causes an exacerbation of DSS-induced colitis. The negative effect of this combinatory approach is the first to be described. During the course of our investigation we discovered that in steady state, CD103^+^ DCs express higher levels of IFNAR1 than CD103^−^ DCs, indicating that Type I IFN signaling may be particularly important for this cell type. Additionally, surface expression of IFNAR1 on CD103^+^ DCs is sensitive to treatment with La-IFN-β and may correlate with induction of Tregs. Overall, the combination of *Lactobacillus* with the secreted IFN-β in our model resulted in the exacerbation of colitis. This study highlights the importance of why the selection of therapeutics delivered by a bacterial vehicle must take into consideration the immunological effects of the vehicle itself.

## Supporting Information

Figure S1 Histogram showing increasing phosphorylation of STAT1 in a C57BL/6 macrophage cell line stimulated for 60 min with 0.5, 5, 50, 500 or 1000 U/ml of IFN-β.
(TIF)

Figure S2 (**A**) Western blot showing the presence of IFN-β in the supernatants of La-IFN-β cultures, and absent in La-EV, used for gastric gavaging of mice. Shown is a representative of this quality control performed for all experiments. (**B**) Percent body weight loss of C57BL/6 mice pretreated with PBS, La-EV or La-IFN-β for 3 days and then administered 3% DSS in their drinking water for 7 d. Day 8 weights are plotted separately to show individual mice (n = 10).
(TIF)

Figure S3 (**A**) Gating strategy for CD45^+^ClassII^+^CD11c^+^CD11b^+^ and CD103^+^ and CD103^−^ DCs in the MLNs, PPs and smIELs of wild-type mice. IFNAR1 expression (% of max) is shown for CD103^+^ and CD103^−^ DC subsets. (**B**) RT-PCR for Foxp3 mRNA in the distal colon of colitic mice pretreated with PBS, La-EV or La-IFN-β relative to a no DSS control (RQ = 1). HRPT was used as an endogenous control.
(TIF)

Figure S4 BMDCs from wild-type mice were matured with TNF-α (50 ng/ml), TNF-α + La-EV or TNF-α + La-IFN-β (100:1 ratio, bacteria:BMDCs) for 2 d. The mature BMDCs were then co-cultured with CD3/CD28 (1 µg/ml) activated splenocytes for 7 d (1:2 ratio, BMDCs:splenocytes). The percent of CD11c^+^ClassII^+^ cells were determined for each maturation treatment by flow cytometry. The data is represented as the mean ± SEM. *** p<0.001.
(TIF)
