# Supplementary figures and images for: Localized Delivery of Interferon-β by Lactobacillus Exacerbates Experimental Colitis
Source: PLoS One. 2011 Feb 18;6(2):e16967. doi: 10.1371/journal.pone.0016967 (PMC3041828; doi:10.1371/journal.pone.0016967)

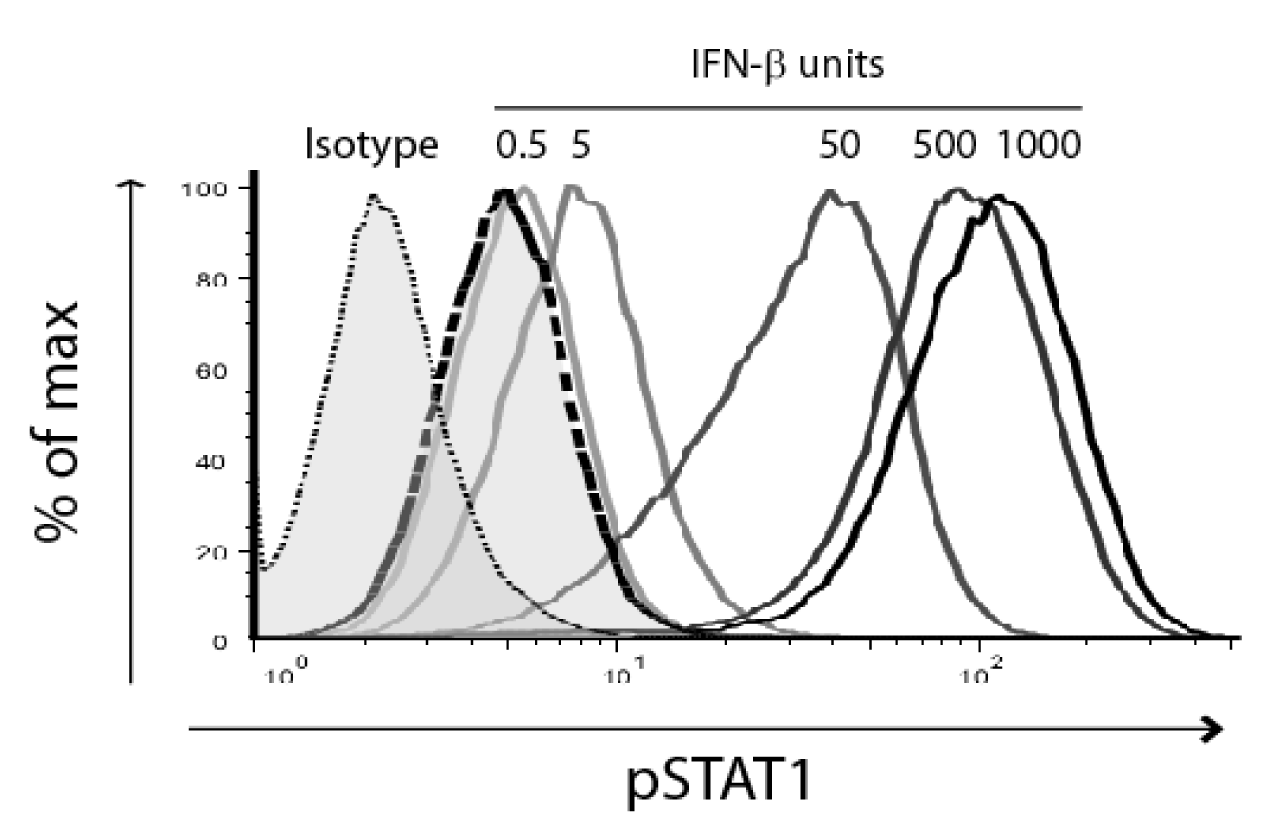

Supplement: Figure S1 — Histogram showing increasing phosphorylation of STAT1 in a C57BL/6 macrophage cell line stimulated for 60 min with 0.5, 5, 50, 500 or 1000 U/ml of IFN-β. (TIF) [file pone.0016967.s001.tif]

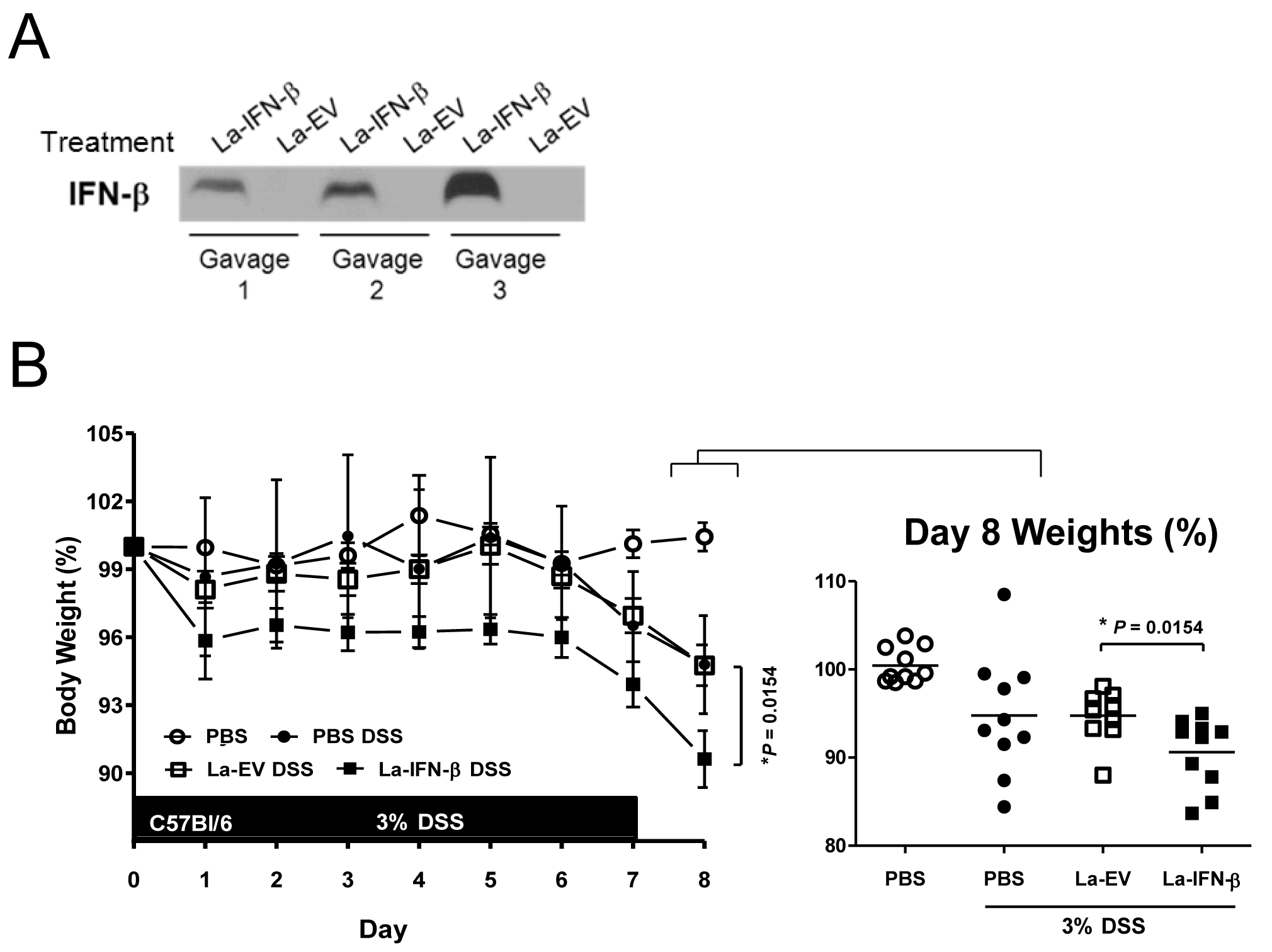

Supplement: Figure S2 — (A) Western blot showing the presence of IFN-β in the supernatants of La-IFN-β cultures, and absent in La-EV, used for gastric gavaging of mice. Shown is a representative of this quality control performed for all experiments. (B) Percent body weight loss of C57BL/6 mice pretreated with PBS, La-EV or La-IFN-β for 3 days and then administered 3% DSS in their drinking water for 7 d. Day 8 weights are plotted separately to show individual mice (n = 10). (TIF) [file pone.0016967.s002.tif]

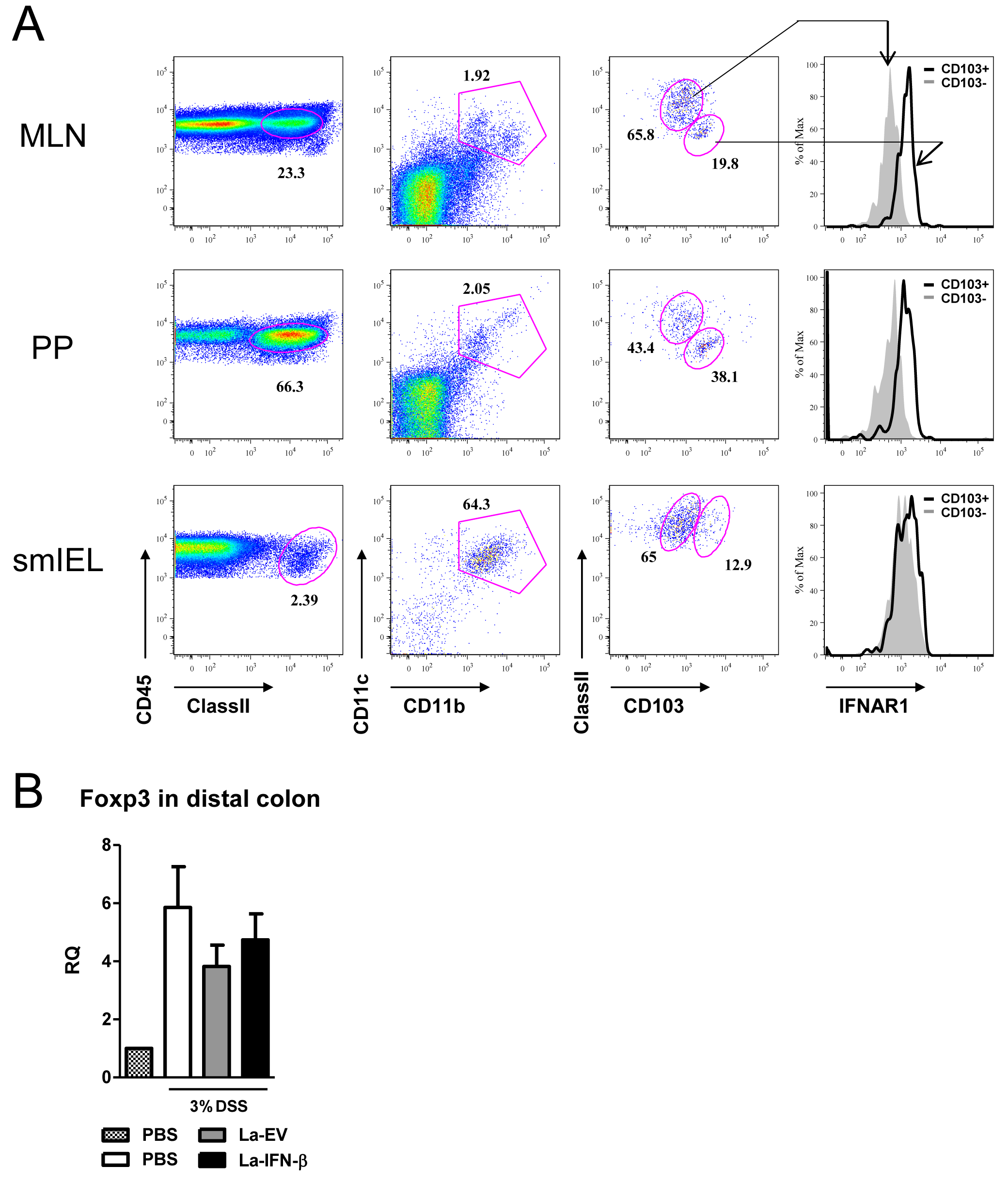

Supplement: Figure S3 — (A) Gating strategy for CD45+ClassII+CD11c+CD11b+ and CD103+ and CD103− DCs in the MLNs, PPs and smIELs of wild-type mice. IFNAR1 expression (% of max) is shown for CD103+ and CD103− DC subsets. (B) RT-PCR for Foxp3 mRNA in the distal colon of colitic mice pretreated with PBS, La-EV or La-IFN-β relative to a no DSS control (RQ = 1). HRPT was used as an endogenous control. (TIF) [file pone.0016967.s003.tif]

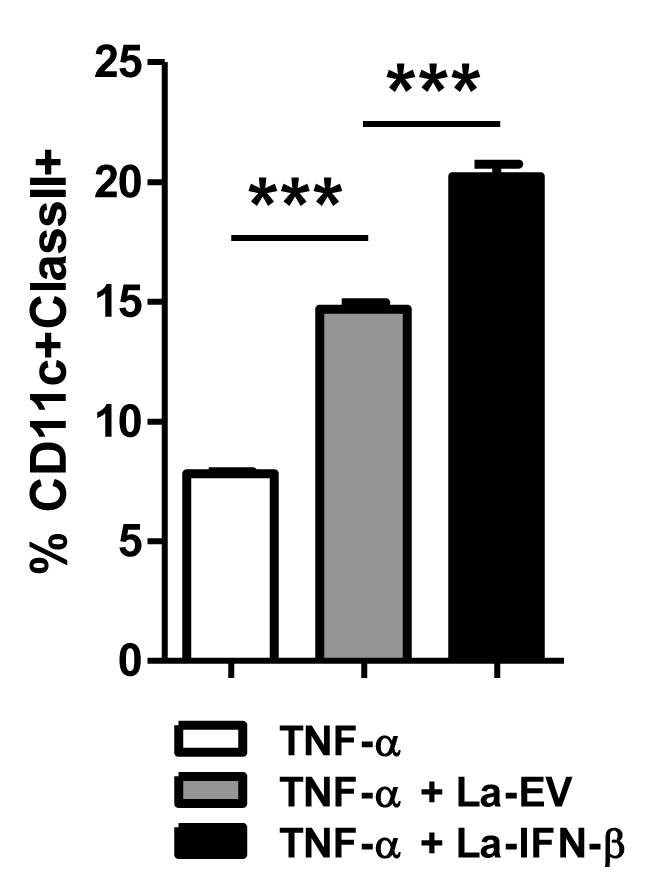

Supplement: Figure S4 — BMDCs from wild-type mice were matured with TNF-α (50 ng/ml), TNF-α + La-EV or TNF-α + La-IFN-β (100:1 ratio, bacteria:BMDCs) for 2 d. The mature BMDCs were then co-cultured with CD3/CD28 (1 µg/ml) activated splenocytes for 7 d (1:2 ratio, BMDCs:splenocytes). The percent of CD11c+ClassII+ cells were determined for each maturation treatment by flow cytometry. The data is represented as the mean ± SEM. *** p<0.001. (TIF) [file pone.0016967.s004.tif]
